# The Impact of Thiamine Treatment in the Diabetes Mellitus

**DOI:** 10.4021/jocmr890w

**Published:** 2012-05-15

**Authors:** Khanh vinh quoc Luong, Lan Thi Hoang Nguyen

**Affiliations:** a Vietnamese American Medical Research Foundation, Westminster, California, USA

**Keywords:** Thiamine, Diabetes Mellitus, Vitamin B_1_

## Abstract

Thiamine acts as a coenzyme for transketolase (T*k*) and for the pyruvate dehydrogenase and α-ketoglutarate dehydrogenase complexes, enzymes which play a fundamental role for intracellular glucose metabolism. The relationship between thiamine and diabetes mellitus (DM) has been reported in the literature. Thiamine levels and thiamine-dependent enzyme activities have been reduced in DM. Genetic studies provide opportunity to link the relationship between thiamine and DM (such as T*k*, *SLC19A2* gene, transcription factor *Sp1*, α-1-antitrypsin, and *p53*). Thiamine and its derivatives have been demonstrated to prevent the activation of the biochemical pathways (increased flux through the polyol pathway, formation of advanced glycation end-products, activation of protein kinase C, and increased flux through the hexosamine biosynthesis pathway) induced by hyperglycemia in DM.Thiamine definitively has a role in the diabetic endothelial vascular diseases (micro and macroangiopathy), lipid profile, retinopathy, nephropathy, cardiopathy, and neuropathy.

## Introduction

Diabetes mellitus (DM) has emerged as a major health problem throughout the world. The prevalence of DM is increasing rapidly in all age groups. The Diabetes Control and Complication Trial (DCCT) and the United Kingdom Prospective Diabetes Study (UKPDS) have demonstrated that intensive treatment showed a delaying in the progression of long-term micro-vascular complications in DM. However, this treatment contributed to a significant increase in severe hypoglycemia [1-2]. Chronic hyperglycemia has been known as a factor in the development of diabetic micro-vascular diseases through increased formation of advanced glycosylation end product (AGE), activation of aldolase reductase (AR) and protein kinase C (PKC), and increased flux through the hexosamine pathway. Benfotiamine, a thiamine derivative, have been demonstrated *in vitro* to counteract the damaging effects of hyperglycemia on cultured vascular cells [3]. Thiamine acts as a coenzyme for transketolase (T*k*) and for the pyruvate dehydrogenase (PDH) and α-ketoglutarate dehydrogenase complexes, enzymes which play a fundamental role for intracellular glucose metabolism by increasing Krebs cycle activity. Therefore, we will review the role of thiamine in the diabetic subjects.

## Relationship Between Thiamine and Diabetes Mellitus

### Nutritional Factor

The relationship between thiamine and DM has been reported in the literature. Significant proportion of healthy subjects (36-47%) was reported as a thiamine-deficient in a hyperglycemic state (such as on a diet high in carbohydrate, diabetes, and pregnancy) [4]. Low plasma thiamine level was noted in type 1 diabetic patients [5]. Thiamine reserve was found to be reduced in litters of untreated diabetic rats [6]. In children, acute thiamine deficiency can be manifested by diabetic ketoacidosis (DKA), lactic acidosis and hyperglycemia [7-8]. In another study, low blood thiamine level, erythrocyte T*k* activity and high erythrocyte thiamine pyrophosphate (TPP) activity have been documented in diabetic patients [9-10]. T*k* has been used to assess thiamine activity in mammalian tissues. The low thiamine values in diabetic patients might be a reduced apo-enzyme level from the disease itself rather than thiamine deficiency [10]. In addition, plasma thiamine level has been shown to be decreased by 76% in type 1 and 75% in type 2 diabetic patients, was associated with increased renal clearance and fractional excretion of thiamine [11]. Furthermore, thiamine transporter protein concentration has been shown to be increased in erythrocyte membranes of type 1 and type 2 diabetic patients. Therefore, changes in thiamine levels may be masked by an increase in thiamine transporter expression.

### Genetic Factor

Genetic studies provide an excellent opportunity to link molecular variations with epidemiological data. DNA sequences variations such as polymorphisms have modest and subtle biological effects.

Thiamine-responsive megaloblastic anemia (TRMA) is a rare autosomal recessive condition, characterized by megaloblastic anemia, non-autoimmune DM, and sensory-neural loss [12-13]. TRMA fibroblasts displayed only 5-10% of thiamine uptake when compared with healthy individuals [14]. In TRMA, DM is heritable and mutation in the SLC19A2 gene on chromosome 1q23.3, which encodes a high-affinity thiamine transport. The result is an abnormal thiamine transportation and thiamine deficiency in the cells. The anemia is corrected with high doses of thiamine and recurred when thiamine is withdrawn. In addition, supplement of high dose of thiamine could improve in the clinical symptoms of the disease including a reduction or cessation in the need for exogenous insulin in these patients [15]. In versa, thiamine withdrawal can lead to DKA in TRMA patients [16]. However, mutations SLC19A2 do not contribute to type 2 DM in Pima Indians [17].

The expression of the genes encoding thiamine transporters (THTR-1 and THTR-2) are regulated via *Sp1* promoter elements [18]. Transcription factor Sp1 mRNA was highly expressed in the epiretinal membrane of the proliferative diabetic retinopathy [19]. *Sp1* inhibitor suppressed transcription of these promoters and inhibited high-glucose-induced mesangial cell proliferation in rat model [20]. Hexosamine pathway has been suggested to participate in the regulation of gene expression by high glucose concentration through *Sp1* DNA binding sites in glomerular mesangial cells [21]. Resistin, an adipocyte-secreted hormone, antagonizes insulin [22]. Overexpression of the resistin gene (*Retn*) in adipose tissue was insulin-resistant in transgenic mice [23], whereas *Retn* (-/-) mice showed lower fasting blood glucose [24]. Osawa et al [25] found that G/G genotype of a resistin polymorphism was associated with type 2 diabetes mellitus by inducing promoter activity through specific binding of *Sp1* and *Sp3* transcription factors.

The α-antitrypsin (ATT) polymorphism with a non-MM genotype has been shown to significantly increased incidence of thiamine deficiency [26]. ATT stimulates insulin secretion and protects β-cells against cytokine-induced apoptosis [27]. Culture of impure human islet fractions in the presence of ATT prevented insulin cleavage and improved islet recovery [28]. In type 1 diabetic mouse model, intradermal human ATT prevents and reverse diabetic disease [29].

Adult bone marrow (BM)-derived insulin-producing cells (IPC) are capable to regulate blood glucose in diabetic mice. Oh et al. [30] demonstrated the presence of T*k* in BM-derived IPCs under high-glucose conditions. Aberrant T*k* has been reported to participate in glucose metabolism in malignant pleural effusion cells [31]. T*k* variants and reduced activities of T*k* enzyme were found in diabetic patients [32-33]. Genetic variability in T*k* and T*k*-like might contribute to the progression of diabetic nephropathy and mortality [34]. A subnormal erythrocyte T*k* activity has been identified in diabetic patients [35]. Thiamine regulates the expression genes that code for enzymes using thiamine as cofactor. Thiamine deficiency diminishes the mRNA levels of T*k* and PDH [36]. PDH activity is reduced in diabetic patients [37-38].

There are numerous potential gene products that are transcriptionally activated by *p53* and are involved in cell cycle arrest or apoptosis [39]. Tumor suppressor *p53* has been identified as a mediator of podocyte (glomerular epithelial cells) apoptosis in cells exposed to high glucose [40]. Glomeruli isolated from these mice showed decreased phosphorylation of AMP-activated protein kinase and enhanced expression of *p53*. High glucose-induced repression of insulin-like growth factor 1 receptor (IGF-1R) is mediated by the association of *p53* with the IGF-1R promoter [41]. Local *p53* silencing resulted in faster wound healing in diabetic wounds [42]. The treated group demonstrated improved wound architecture while demonstrating near-complete local *p53* knockdown. Morimoto et al [43] suggested that *p53* accumulation may be responsible for impaired wound healing in diabetes. Atovastatin was found to restore ischemic limb loss in diabetes by augmenting *p53* degradation. The *p53* codon 72 polymorphism has been reported in type 1 DM [44]. Increased thiamine transporter activities were found in cells over-expressing mTHTR-1 and under conditions of DNA damage or *p53* activation [45]. Thiamine diphosphate (TDP) has been shown to inhibit *p53* binding and thiamine has been shown to inhibit intracellular *p53* activity [46]. The expression of *p53* was decreased significantly in cultured retina neurons of diabetic rats treated with thiamine [47].

## Thiamine and Biochemical Consequences of Hyperglycemia

There are four distinct biochemical pathways, which have been identified as mechanisms by which intracellular hyperglycemia can induce vascular damage and contribute to the pathogenesis of diabetic complications: increased flux through the polyol pathway, formation of AGE, activation of PKC, and increased flux through the hexosamine biosynthesis pathway (HBP).

Thiamine and benfotiamine reduces AR mRNA expression, activity, sorbitol concentrations, and intracellular glucose while increasing the expression and activity of T*k* in human endothelial cells and bovine retinal pericytes cultured in high glucose [48]. AR is a key enzyme in the polyol pathway, which transforms D-glucose into D-sorbitol.

In experimental diabetes, thiamine and benfotiamine (a synthetic S-acyl derivative of thiamine) supplement prevented tissue accumulation and increased urinary excretion of protein glycation, oxidation and nitration adducts [49]. Karachalias et al [50] reported that hydroimidazolone AGE residues derived from glyoxal and methylglyoxal, G-H1 and MG-H1, were increased 115% and 68% in the streptozotocin-induced (STZ) diabetic rats, and were normalized by both thiamine and benfotiamine; whereas N-carboxymethyl-lysine (CML) and N-carboxyethyl-lysine (CEL) residues were increased 74% and 118% in diabetic-induced rats and were normalized by thiamine only.

High glucose has been reported to increase diacylglycerol mass and activates PKC in mesangial cell cultures [51]. High dose of thiamine and benfotiamine increased T*k* expression in renal glomeruli and associated with decreased activation of PKC and also decreased protein glycation and oxidative stress [52].

*O*-glycosylation of protein induced by HBP activation has been reported to modify collagen expression and contribute to the diabetic cardiomyopathy [53-54]. Thiamine replacement decreased *O*-glycosylation of protein and prevented diabetes-induced cardiac fibrosis in experimental diabetes [55].

## Role of Thiamine in the Diabetes Mellitus

### Blood Glucose and Insulin Secretion

When rats were maintained on a thiamine-deficient diet, cerebral glucose utilization (CGU) decreased as the concentration of cerebral thiamine diminished [56]. The addition of thiamine reversed changes in the CGU resulting from thiamine deprivation [57]. High fiber diets have been reported to decrease postprandial glycemia in diabetic patients [58]. Thiamine is present in high amounts in fiber-containing foods [59]. In women, the effect of thiamine intake appeared to have a strong and revelant association with glucose tolerance [60]. In a randomized controlled trial, thiamine has showed to decrease blood glucose and leptin concentration in 24 drug-na?ve patients with diabetes type 2 in one month [61]. Glycosylated hemoglobin significantly decreased with benfotiamine treatment in 45 days [62].

The pancreas contains high levels of thiamine [63]. In pancreas, thiamine uptake is carrier mediated and is adaptively regulated by the prevailing vitamin level via transcriptional mechanisms [64]. Thiamine deficiency leads to a marked impairment in insulin synthesis and secretion [65-66]. Rats with thiamine-deficient diet caused the reduction of serum insulin by 14%, and also decreased trans-membrane glucose transport [66]. Benfotiamine activates glucose metabolism and insulin synthesis to prevent glucose toxicity caused by hyperglycemia in DM [30]. This vitamin B also appeared to maximize the BM-derived IPCs ability to synthesize insulin [30].

### Endothelial Dysfunction

Dysfunction of endothelial cells has been known to play a major role in both micro- and macrovascular complications of DM. Thiamine reversed hyperglycemia-induced dysfunction in cultured endothelial cells [67]. Thiamine and benfotiamine have been demonstrated *in vitro* to counteract the damaging effects of hyperglycemia on cultured vascular cells [68]. In addition, thiamine has been reported to improve endothelia vasodilatation in patients with hyperglycemia [69]. Daily intake of thiamine was positively correlated with the circulating level of endothelial progenitor cells and vascular endothelial function in type 2 diabetic patients [70].

### Cardiovascular Disease

The most common cause of morbidity and mortality among diabetic patient is atherosclerotic cardiovascular disease. In diabetic-induced mice with unilateral limb ischemia, benfotiamine prevented ischemia-induced toe necrosis, improved hindlimb perfusion and oxygenation, and restored endothelium-dependent vasodilation. Histological studies revealed the improvement of reparative neovascularization and inhibition of endothelial and skeletal muscle cells apoptosis [71].

Diabetic cardiomyopathy can progress toward overt heart failure with increased mortality. Benfotiamine improved functional recovery of the infarcted heart with prolonged survival and reduced cardiomyocyte apoptosis in diabetic mice [72]. High dose of thiamine rescues cardiomyocyte contractile dysfunction and it also prevented diastolic dysfunction, heart failure and cardiac fibrosis in diabetes-induced mice models [73-74, 54].

### Lipid Profiles

Cardiovascular disease in diabetes is linked to increased risk of atherosclerosis, increased levels of triglyceride-rich lipoproteins and enhances hepatic lipogenesis. High dose of thiamine therapy (70 mg/kg) prevented increased in plasma cholesterol and triglycerides in diabetes-induced rats but it did not reverse decrease of HDL [75]. However, a lower dose of thiamine (7 mg/kg) and the benfotiamine were ineffective in preventing these lipid profiles [76].

### Nephropathy

Nephropathy is a common complication of diabetes. It is characterized by the development of proteinuria and end-stage renal disease (ESRD). In diabetic rat model, high-dose of thiamine and benfotiamine strongly inhibited the development of micro-albuminuria and associated with decreased activation of PKC, protein glycation, and oxidative stress [59]. This was achieved without change in elevated plasma glucose concentration and glycated hemoglobin. In type 2 diabetic nephropathy, a high dose of benfotiamine (900 mg/day) treatment did not reduce the urinary albumin excretion (UAE) and the tubular damage marker kidney injury molecule-1 (KIM-1) after 12 weeks [77]; this study may have been too short to see the effect of benfotiamine. This vitamin prevented oxidative stress induced by the mutagen 4-nitroquinoline-1-oxide (NQO), the uremic toxin indoxyl sulfate, and the peptide hormone angiotensin II in three different kidney cell lines [78]. In a double-blind placebo-controlled study, urinary albumin excretion was decreased in type 2 diabetic patients with micro-albuminuria after receiving high-dose of thiamine for 3 months [79]. In another study with high dose of thiamine, the level of urinary albumin decreased by 34% in the type 2 diabetic patients [80].

### Neuropathy

Diabetes polyneuropathy is one of the most common diabetic complications. Benfotiamine has shown to effect in the diabetic neuropathy patients with reduction in pain score and improving neurophysiological parameters [47]. Benfothiamine significantly reduced inflammatory (10 - 300 mg/kg) and neuropathic (75 - 300 mg/kg) nociception in non-diabetic and diabetic rats [81]. In a double blind, placebo-controlled, phase-III clinical study with benfotiamine in diabetic polyneuropathy, the improvement of neuropathy symptom score was more pronounced at the higher benfotiamine dose (600 mg vs. 300 mg) and increased with treatment duration [82].

High-dose thiamine treatment may be beneficial in delaying the progression of diabetic cystopathy in the experimental animal model [82].

### Retinopathies

Diabetic retinopathy is one of the most serious complications in diabetic patients and a leading cause of blindness. Polyol pathway hyperactivity has been implicated in the pathogenesis of diabetic retinopathy [83-85]. T*k* has been demonstrated to be a structural protein in cornea, being important for eye transparency [86]. T*k* is also known as an important role in preventing hyperglycemia-induced vascular damage [53]. Thiamine and benfotiamine were reported to regulate the intracellular glucose and polyol pathway in bovine retinal pericytes cultured in high glucose [53]. Early and selective loss of pericytes and thickening of the basement membrane are hallmarks of diabetic retinopathy. Thiamine and benfotiamine prevent apoptosis induced by high glucose-conditioned extracellular matrix in human and bovine retinal pericytes (HRP and BRP) [77-87]. These vitamins B correct the increase in matrix metalloproteinase 2 (MMP-2) activity due to high glucose in HRP, while increasing their tissue inhibitors (TIMP-1) [87]. In retinas of diabetic animals, benfotiamine treatment inhibited these three pathways and NF-kappaB activation by activating T*k*, and also prevented experimental diabetic retinopathy [3].

### Cancer

Epidemiological data have suggested an increased cancer rates in diabetic patients [88]. Diabetes or hyperglycemia causes DNA damage by oxidation to bases and the sugar-phosphates has been demonstrated recently [89-91]. High level of glucose also reported to increase mutagenesis in human lymphoblastoid cells [92]. However, benfotiamine significantly lowered the genomic damage in peripheral lymphocytes of hemodialysis patients [93]. It also exhibits direct anti-oxidative capacity and prevents induction of DNA damage *in vitro* [77].

## Conclusion

The relationship between thiamine and diabetes mellitus was discussed. Thiamine definitively has a role in the diabetic endothelial vascular diseases (micro and macroangiopathy), lipid profile, retinopathy, nephropathy, cardiopathy, and neuropathy.
